# Integrating Machine Learning into Statistical Methods in Disease Risk Prediction Modeling: A Systematic Review

**DOI:** 10.34133/hds.0165

**Published:** 2024-07-23

**Authors:** Meng Zhang, Yongqi Zheng, Xiagela Maidaiti, Baosheng Liang, Yongyue Wei, Feng Sun

**Affiliations:** ^1^ Department of Epidemiology and Biostatistics, School of Public Health, Peking University, Beijing, China.; ^2^ Key Laboratory of Epidemiology of Major Diseases (Peking University), Ministry of Education, Beijing, China.; ^3^ Peking University First Hospital, Beijing, China.; ^4^ Department of Biostatistics, School of Public Health, Peking University, Beijing, China.

## Abstract

**Background:** Disease prediction models often use statistical methods or machine learning, both with their own corresponding application scenarios, raising the risk of errors when used alone. Integrating machine learning into statistical methods may yield robust prediction models. This systematic review aims to comprehensively assess current development of global disease prediction integration models. **Methods:** PubMed, EMbase, Web of Science, CNKI, VIP, WanFang, and SinoMed databases were searched to collect studies on prediction models integrating machine learning into statistical methods from database inception to 2023 May 1. Information including basic characteristics of studies, integrating approaches, application scenarios, modeling details, and model performance was extracted. **Results:** A total of 20 eligible studies in English and 1 in Chinese were included. Five studies concentrated on diagnostic models, while 16 studies concentrated on predicting disease occurrence or prognosis. Integrating strategies of classification models included majority voting, weighted voting, stacking, and model selection (when statistical methods and machine learning disagreed). Regression models adopted strategies including simple statistics, weighted statistics, and stacking. AUROC of integration models surpassed 0.75 and performed better than statistical methods and machine learning in most studies. Stacking was used for situations with >100 predictors and needed relatively larger amount of training data. **Conclusion:** Research on integrating machine learning into statistical methods in prediction models remains limited, but some studies have exhibited great potential that integration models outperform single models. This study provides insights for the selection of integration methods for different scenarios. Future research could emphasize on the improvement and validation of integrating strategies.

## Introduction

Disease risk prediction uses multiple predictors to estimate the risk of disease onset at a specific time or the probability that a clinical outcome will occur in an individual [1]. The prediction results furnish invaluable insights into diagnosis and prognosis for clinicians, patients, and healthcare providers, contributing greatly to the timely identification, intervention, and management of diseases. As such, disease prediction has become an important topic in the field of medicine [2]. Clinical prediction models include diagnostic model and prognostic model [3]. The diagnostic model predicts the presence or absence of a disease or outcome based on an individual’s current state [4]. The prognostic prediction model centers on the probability of a specific outcome occurring in the future, based on an individual’s current health status [5,6]. Some clinical guidelines recommended the application of prediction models in clinical diagnosis and treatment, aiming to promote more efficient and personalized clinical decision-making [7,8].

The statistical model is a mathematical representation of statistical assumptions about how observable data are generated [9], which is commonly used for disease risk prediction in the medical field. Traditional statistical models mainly include logistic regression (LR) for binary responses, Cox proportional hazards regression for survival outcomes, linear regression for continuous outcomes, and Poisson regression for count outcomes [9,10]. Statistical models have well-defined statistical inference processes, simplicity in implementation, and good interpretability; thus, they have important clinical value and wide application. However, the model assumptions of these statistical methods may not hold in practice, causing potential model misspecification and hence resulting in bias or even misleading results in disease prediction.

With the development of artificial intelligence, its branch machine learning has created new reliable and effective methods, promoting the use of unstructured data and longitudinal data for disease prediction. Machine learning focuses on developing data-driven approaches for computers to learn and improve their performance [11,12]. This method has relatively low requirements and restrictions on data structure and model assumptions, so it is more flexible. Machine learning makes superior predictions compared to statistical methods in some cases [10,13]. However, a recent systematic review found no evidence of superior performance of machine learning over LR [14]. It is important to note that statistical models and machine learning actually have a certain overlap, and the distinction between the two has still been unclear. Considering that one definition of machine learning is that it focuses on models that directly and automatically learn from data [15], LR does not fall under machine learning using this definition. Therefore, LR was classified as a statistical model in this study.

In reality, sometimes we may not know which method is most suitable for the applied data in different scenarios. A single type of algorithm is not always suitable for identifying all different potential patterns in big data. Misuse of models could lead to diminished prediction accuracy and increased susceptibility to wrong prediction. Previous studies have found that constructing and combining multiple models can achieve better performance than a single model [16]. Integration models use multiple trained models together to give a final prediction result through different ways. The effective integration of machine learning into traditional statistical methods may improve the precision and accuracy of clinical decision-making and help to establish prediction models with generalization and stability within multifaceted contexts. In addition, different kinds of variables can be integrated using various methods through integration models, minimizing computational complexity while fully leveraging clinical data. However, the existing research lacks insight into the current methodologies and corresponding specific application scenarios of the integration of machine learning and statistical methods in disease risk prediction modeling.

To address this gap, this study conducts a systematic review of disease prediction models, which integrate machine learning into statistical methods. Through this study, we analyze the characteristics, modeling details, and application scenarios of integration models. By doing so, we aim to provide future directions for selecting optimal integration modeling methodologies in diverse scenarios, fostering the advancement of clinical diagnosis and screening practices.

## Methods

Three English databases (PubMed, Embase, and Web of Science) and 4 Chinese databases (CNKI, VIP, WanFang, and SinoMed) were searched from database inception to 2023 May 1st. The search strategy utilized a combination of Chinese and English search terms related to essential concepts including “Artificial intelligence,” “Machine learning,” “Ensemble learning,” “Disease prediction,” “Risk prediction,” “Prediction model,” “Diagnosis model,” and so on. Detailed search strategies of each database were shown in the Supplementary Materials. The search results were imported into EndNote X9, and duplicates were removed automatically using the software. The titles and abstracts of each search results were carefully reviewed based on predefined inclusion and exclusion criteria. Seemingly eligible full-text researches were then thoroughly read to make the final determination of inclusion or exclusion in the review.

Inclusion criteria were as follows: (a) studies constructing new models for disease diagnosis, prediction of disease occurrence, or prognosis (outcomes like death, disability, disease recurrence, complications, or treatment response) and (b) investigations involving the integration of traditional statistical methods (LR, Cox proportional hazards regression, Poisson regression, linear regression, etc.) and machine learning.

Exclusion criteria were as follows: (a) studies that were not relevant with disease prediction; (b) studies only using statistical methods or machine learning, or only comparing the two; (c) letters, editorials, commentaries, advertisements, agency reports, website announcements, etc.; (d) duplicated studies and papers without full text; and (e) studies not in English or Chinese.

Data extraction was conducted in Microsoft Excel. The information extracted contained the following: (a) basic information: the first author, publication year, journal information, training data type, data source, target population, outcome, prediction interval or follow-up time, total sample size, sample size of outcome occurring, and the ratio of the sample size of the training set to the internal validation set; (b) integration-related content: the statistical method used, machine learning utilized, names of the integration models, and types and specifics of integrating methods; (c) comparison between integration models and single methods: criteria used for model comparison and their values, such as area under the receiver operating characteristic curve (AUROC), accuracy, sensitivity, specificity, and *F*-measure, results of model comparison, and the presence of external validation; and (d) modeling-related content: number of candidate predictors, dimensions of candidate predictors, number of final predictors included in the model, data missing, and processing of missing value. Since many included studies focus on integration methods and do not provide detailed information about participants, predictors, and other relevant content, the Prediction model Risk Of Bias ASsessment Tool (PROBAST) was not applicable. Therefore, quality assessment was not conducted in this study.

Both the study selection and data extraction were performed independently and concurrently by 2 researchers. Any discrepancies arising during screening or data extraction process were resolved through discussion to achieve consensus and determine the final outcome of the review.

For studies that employed multiple machine learning algorithms, we only presented the performance of best-performing algorithm. When a study utilized multiple validation datasets, we displayed the median of this metric across different datasets. SPSS 26.0 software was used to analyze the data. Nonparametric test was used to compare continuous variables that do not satisfy the normal distribution, with statistical significance indicated by 2-tailed *P* < 0.05.

## Results

**Fig. 1.**
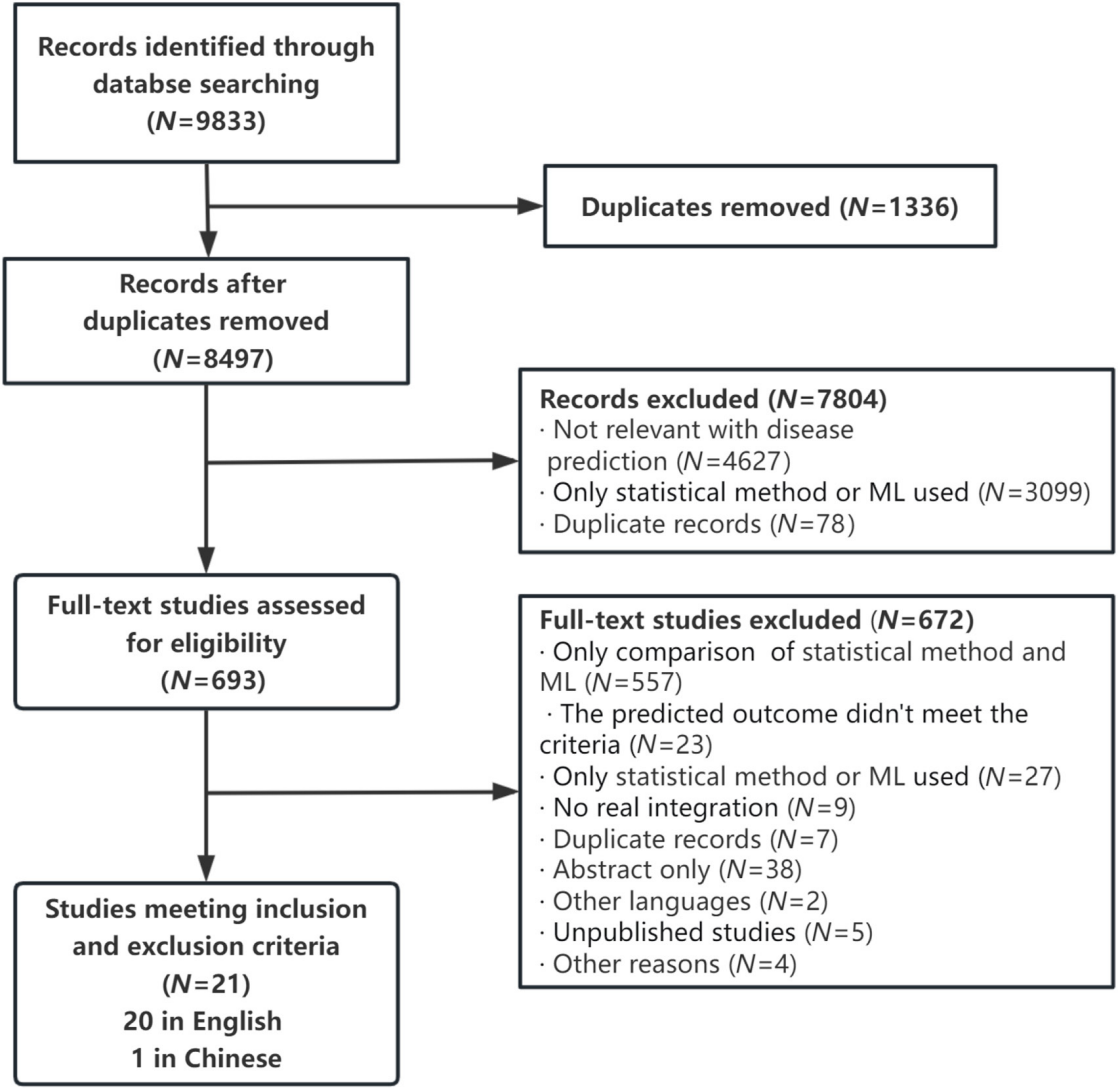
Flow diagram for study selection. ML, machine learning.

As shown in Figure. 1, the initial search yielded a total of 9,833 records. After selection, 20 English and 1 Chinese study centered on integrating machine learning into statistical methods were included [17–37]. The first study was published in 1997, and the number of related studies has increased rapidly since 2021. Among these, 5 studies were about the diagnostic model, while others predicted disease occurrence or prognosis. Specifically, 4 studies focused on predicting disease occurrence, 6 studies on predicting prognosis, and other 6 studies on assessing mortality risk. Majority of data types are multi-centric (61.1%, 11/18). The data source mainly consists of data from hospitals, public databases, and national registration data. The predicted outcomes were primarily about cancer (including lung, colorectal, and breast cancer) as well as chronic diseases such as cardiovascular disease, stroke, and type 2 diabetes. The prediction time interval ranged from a minimum of 48 h to a maximum of 10 years (see Table S1).

The models mainly include 2 categories: classification (the final output is a categorical variable) and regression (the final output is a continuous variable) [38]. As shown in Table 1, the integrating strategy of regression models mainly consisted of 3 categories. The first method, simple statistics, was simple statistical aggregation of output from different methods, including averaging predicted probabilities or selecting the maximum value. The second category, weighted statistics, was built upon the first method by incorporating weight, such as using metrics like the *C*-statistic or calibration errors to determine model weight, and ultimately yielded a normalized risk probability output. The third category, named as stacking model, constituted a large proportion of integration models. Models constructed using this method generally consisted of 2 or more layers [39]. In the first layer, some statistical methods and/or machine learning algorithms were used for predictions. The results of these predictions were then input into the second layer for final predictions. The second layer of stacking commonly employed LR or XGBoost.

**Table 1.** The studies included and types of integration related to regression model

First author, publication year	Statistical methods	Machine learning	Types of integrating	Ways of integrating	Comparison of performance	External validation
Lippmann and Shahian [17]	LR	MLP	Simple averaging	The output was derived from a simple average of the outputs of LR and MLP classifiers.	Integration model outperformed other models.	No
Lv and Wang [18]	LR	AdaBoost; Class balanced SVM	Simple averaging	The results of the 3 classifiers were arithmetically averaged to obtain the final output.	Integration model outperformed other models.	Yes
Dessie et al. [19]	LR	SVMR; KNN; NN	Simple averaging	The integration model used the average predicted risk probabilities of LR, SVMR, KNN, and NN.	Integration model outperformed other models.	Yes
Pai et al. [20]	LR	XGBoost; RF; CNN	Simple averaging or maximum	Based on the average probability or maximum probability: XGB + CNN, RF + CNN, LR + CNN, XGB + RF + LR + CNN.	Integration model outperformed other models.	No
Basu and Narayanaswamy [21]	LR with elastic net regularization	RF; GBM; DNN	Weighted combination of all the models	The final integration prediction was given by the weighted average of the predictions from each base model to maximize the *C*-statistic in repeated cross-validation samples from the training data.	RF outperformed other models.	No
Gao et al. [22]	LR	SVM; GBDT; NN	Weighted combination of all the models	The mortality risk probability of each individual estimator (LR, SVM, GBDT, and NN) was integrated by manually assigning weights with 0.25, 0.3, 0.1, and 0.35, respectively.	Integration model outperformed other models.	Yes
Torquati et al. [23]	LR	GAM; XGBoost	Weighted combination of all the models (based on the negative log-likelihood loss)	The weighted combination of main terms LR, stepwise LR, GAM, and XGBoost was calculated using the negative log-likelihood loss and 5-fold sample splitting.	Integration model outperformed other models.	No
Warden et al. [24]	Penalized LR	RF	Stacking (LR as the meta-classifier)	The integration model fit a penalized LR that used the probability generated by the final RF as a predictor.	Integration model outperformed other models.	No
Zeng et al. [25]	LR	LDA; CART; NB; KNN; MLP; SVM; RF; XGBoost	Stacking (LR as the meta-classifier)	LR used the predicted probabilities of 9 base model predictions as input and generated the output of the stacking model.	Integration model outperformed other models.	Yes
Khera et al. [26]	LR	XGBoost; NN	Stacking (XGBoost as the meta-classifier)	Th risk estimates of LR, XGBoost, and NN were used to train the level 2 XGBoost classifier and generated the final output.	Compared with LR, the integration model had a higher AUROC (the difference was not statistically significant), with more precise classification across the risk spectrum.	No
Liu et al. [27]	Cox regression	XGBoost	Stacking (XGBoost as the meta-classifier)	Based on Cox model, log HR was used as input of XGBoost, and XGBoost was further optimized.	Integration model outperformed other models.	No
Liu et al. [28]	LR	CNN	Stacking (LR as the meta-classifier)	Predictors with *P* < 0.05 screened out by univariate LR analysis were combined with the CNN model score and included in a multivariate LR model.	Integration model outperformed other models.	Yes
Lee et al. [29]	LR	MLP; RF; SVM; KNN; XGBoost; CART; Bayes	Stacking (LR as the meta-classifier); Averaging	1. Stacking: LR used the output of MLP, RF, SVM, KNN, XGBoost, CART, and Bayes and generated the output. 2. The output was derived from an average of the outputs of all the models.	LR outperformed other models.	No
Fan et al. [30]	LR	NB; RF; SVM; FNN	Stacking (LR as the meta-classifier); Simple averaging, Weighted averaging	LR used the output of 5 base models and generated the output. In weighted averaging, the weight of each base model was set to the reciprocal of the expected calibration error and maximum calibration error.	Integration model outperformed other models.	No

LR, logistic regression; MLP, multi-layer perceptron; SVM, support vector machine; SVMR, support vector machine with the radial kernel; KNN, K-nearest neighbor; NN, neural network; XGBoost, eXtreme gradient boosting; RF, random forest; CNN, convolutional neural networks; GBM, gradient boosting machines; DNN, deep learning neural network; GBDT, gradient boosting decision tree; GAM, generalized additive models; LDA, linear discriminant analysis; CART, classification and regression trees; NB, naïve Bayes

The integration methods were used for incorporating some high-dimensional variables (genes, image characteristics, prescription medications code) or highly collinear variables (social determinants of health) while using traditional clinical predictors for modeling. Comparing 3 integration methods, the median sample sizes for studies using only one of the 3 methods were 1,351 (simple statistics), 398,833 (weighted statistics), and 12,558 (stacking), respectively, but the differences among the methods were not statistically significant (*P* > 0.05). In terms of the number of final predictors, stacking [with eXtreme Gradient Boosting (XGBoost) as the meta-classifier] was used for cases with more than 100 predictors included. The majority of studies had low proportion (<5%) of missing values for the predictors (see Table 2).

**Table 2.** Modeling details in the studies of regression model

First author, publication year	Total sample size (outcomes)^a^	Training: internal validation	Number of candidate predictors	Dimensions of candidate predictors^b^	Number of final predictors	Data missing	Processing of missing value	Types of integrating
Lippmann and Shahian [17]	40,480 (1,386)	1: 1	59	Demographics, PA, MH, comorbidities, DC, medications	36	1 variable with missingness >5%	Impute missing values to the mode for categorical variables and median for continuous variables	Simple averaging
Lv and Wang [18]	1,125 (107)	NA	12	Demographics, DC, LT	12	NA	NA	Simple averaging
Dessie et al. [19]	501 (180)	10-fold-validation	745	Survival-associated differentially expressed genes	9	No missing	NA	Simple averaging
Pai et al. [20]	1,577 (383)	5-fold-validation	NA	PA, LT, ventilatory parameters, image	21 clinical features + image feature	NA	NA	Simple averaging or maximum
Basu and Narayanaswamy [21]	1,015,808 (152,558)	3: 1	NA	Demographics, LB, MH, comorbidities, LT, social determinants of health	35 (LR); 37 (RF)	NA	No imputation of missing data	Weighted combination
Gao et al. [22]	1,243 (175)	1: 1	53	Demographics, PA, LB, comorbidities, LT, image	14	19 variables with missingness >5%	R-package missForest	Weighted combination
Torquati et al. [23]	398,833 (15,380)	5-fold-validation	56	Demographics, PA, LB, MH, comorbidities, medications	56	NA	Patients with missing data were excluded	Weighted combination
Warden et al. [24]	88,265 (35,941)	9: 1	994	Demographics, LB, prescription medications code, number of unique diagnosis codes	194 (LR-without medications); 270 (LR-including medications); 272 (RF)	NA	NA	Stacking (LR as the meta-classifier)
Zeng et al. [25]	12,558 (2,253)	5-fold-validation for adjusting hyperparameter	65	Demographics, PA, LB, LT, comorbidities, DC, therapy	34 (way 1); 23 (way 2); 12 (way 3)	NA	KNN imputation	Stacking (LR as the meta-classifier)
Khera et al. [26]	755,402 (33,238)	3: 1	NA	Demographics, PA, MH, comorbidities, LT, medications, electrocardiogram	29 (way 1); 56 (way 2)	1 variable with missingness >5%	Exclude covariates with missingness >5%. Impute missing values to the mode for categorical variables and median for continuous variables. Sensitivity analyses: MICE	Stacking (XGBoost as the meta-classifier)
Liu et al. [27]	4,575 (5 y: 616; 10 y: 933)	7: 3	89	Demographics, LB, MH, DC, pregnancy-related features, therapy	24	Patients with ≥ 8 variables missing accounted for 24%	Patients with the number of missing fields ≥ 8 are excluded	Stacking (XGBoost as the meta-classifier)
Liu et al. [28]	CNN: 3,644 (3,644); hybrid: 756 (501)	NA	12	Demographics, LB, MH, family history, comorbidities, exposure history, image	5	No missing	NA	Stacking (LR as the meta-classifier)
Lee et al. [29]	3,690 (738)	8: 2	74	MH, comorbidities	32 (way 1); 19 (way 2); 9 (way 3)	No missing	NA	Stacking; Averaging
Fan et al. [30]	406 (116)	8: 2 and 3-fold cross-validation	17	Demographics, family history, LT, DC, medications	5	No missing	NA	Stacking; Simple averaging; Weighted averaging

PA, physical examination; MH, medical history; DC, disease characteristics; LT, laboratory tests; LB, living habit; MICE, multiple imputation by chained equations

^a^ Total sample size refers to the sample size of the training set plus the sample size of the internal validation set, excluding the external validation data.

^b^ Demographics (age, gender, race, nationality), living habits (smoking, alcohol consumption, exercise routine, diet), physical examination (height, weight, body mass index, body temperature, respiration, pulse, blood pressure), and disease characteristics (such as tumor grading and staging).

Similar to the regression models, a total of 7 studies were included for classification prediction models, which predominantly encompassed 4 integrating approaches (Table 3). The first approach was majority voting, which aggregated the votes that related to each class label and output the one with the most votes as a candidate class. The second method, weighted voting, based on voting, involved weights. The weights of various models were determined using performance indicators like *F*-measure [32]. This approach considers both the classifications of each model and their respective weight sums. The final choice of class was determined based on the results of the sum for different classes. The third method was stacking. The fourth method, model selection, first employed statistical methods and machine learning for predictions. For individuals where the predictions of the 2 methods diverged, a new method, such as decision tree, was used to select between statistical method and machine learning [37]. The sample sizes used in the latter 2 methods were higher than the former 2 methods. The fourth method was applied to the modeling of more than 50 final predictors. The majority of studies also had a low proportion of missing values. When there were missing data, for continuous variables, missing values were mainly imputed using the means, while for categorical variables, missing values were considered as a special category. For majority voting method, the ratio between the number of outcome events and the number of features expanded by convolutional neural networks (CNN) was much less than 10 (Table 4).

**Table 3.** The included studies and types of integration related to classification model

First author, publication year	Statistical methods	Machine learning	Types of integration	Ways of integration	Comparison of performance	External validation
Rustam et al. [31]	LR	SGDC; SVM	Majority voting	LR, SVM, and SGDC were joined using the majority voting criteria.	Integration model outperformed other models.	Yes
Bashir et al. [32]	Linear regression	NB; QDA; IBL; SVM	Weighted voting (*F*-measure as the weight)	Weights were normalized by min–max normalization using *F*-measure. According to the final classification of each classifier and their weight, determine the final addition results of different classes and which class to choose.	Integration model outperformed other models.	Yes
Wu et al. [33]	LR	K-means	Stacking (LR was used in the second level)	In the first level, the improved K-means was used to remove incorrectly clustered data and the optimized dataset was input for next level. Then, LR was used to classify the remaining data.	Integration model outperformed other models.	Yes
Kong et al. [34]	LR	SVM; RF; KNN	Stacking (LR as the meta-classifier)	LR used the outputs of SVM, RF, and KNN as input and generated the output of the stacking model.	Integration model outperformed other models.	No
Zhang et al. [35]	LR	GBDT; RF; AdaBoost; XGBoost; NB; DT; MLP	Stacking (LR as the meta-classifier)	LR used the outputs of 8 base model predictions containing LR as input and generated the output of the stacking model.	Integration model outperformed other models.	No
Dritsas and Trigka [36]	LR	NB; RF; RepTree; J48	Stacking (LR as the meta-classifier); Majority voting	Stacking: NB, RF, RepTree, and J48 were used as base classifiers, whose predictions were used to train LR. Majority voting: This method aggregated the votes that relate to each class label and output the one with the most votes as a candidate class.	Stacking was the optimal algorithm, followed by RF and Majority voting	No
Chun et al. [37]	Cox regression	GBT; DT	New method was used to select the final model when 2 models disagreed.	A second training set was generated by restricting the validation set to the individuals for whom the Cox model and GBT disagreed, and a decision tree was derived from this training set	Integration model outperformed other models.	No

SGDC, stochastic gradient descent classifier; QDA, quadratic discriminant analysis; IBL, incremental Bayesian learning; DT, decision tree; GBT, gradient boosted trees

**Table 4.** Modeling details in the studies of classification model

First author, publication year	Total sample size^a^	Training: Internal validation	Number of candidate predictors	Dimensions of candidate predictors^b^	Number of factors eventually included	Data missing	Processing of missing value	Types of integration
Rustam et al. [31]	918 (508)	8: 2	11	Demographics, DC, electrocardiogram, LT	11; 25,000 (expanded from the CNN)	NA	NA	Majority voting
Bashir et al. [32]	267 (NA); 267 (NA); 303 (NA); 270 (NA); 209 (NA)	10-fold-validation	22; 44; 14; 14; 8	Demographics, DC, electrocardiogram, LT	22; 44; 14; 14; 8	NA	Remove variables with missingness ≥50%; for variables with missingness <50%, impute missing values to group mean	Weighted voting
Wu et al. [33]	768 (268)	10-fold-validation	8	Demographics, PA, pregnancy-related features, LT	8	NA	Impute missing values to the mean	Stacking (LR was used in the second level)
Kong et al. [34]	23,992 (7,270)	5-fold-validation	NA	Demographic, comorbidities, admission-related features	15	2 variables with missingness <15%	Missing values were considered as a special category	Stacking (LR as the meta-classifier)
Zhang et al. [35]	1,045 (766)	8: 2	NA	Demographics, PA, LT, DC	4 (perform best)	5 variables with missingness, and the percentage of missing <5%	KNN	Stacking (LR as the meta-classifier)
Dritsas and Trigka [36]	3,254 (180)	10-fold-validation	10	Demographics, PA, LB, comorbidities, LT	10	No missing	NA	Stacking (LR as the meta-classifier); Majority voting
Chun et al. [37]	503,842 (9 y: 43,234)	85% (training); 12.75% (validation); 2.25% (test)	143	Demographics, PA, LB, family history	Cox: 66 (male); 70 (female)	16 variables with missingness, and the percentage of missing <5%	Impute missing values using the means in the training set. 3 binary risk factors were added to represent whether or not an individual was missing.	New method was used to select the final model when 2 models disagreed.

^a^ Total sample size refers to the sample size of the training set plus the sample size of the internal validation set, excluding the external validation data.

^b^Demographics (age, gender, race, nationality), living habits (smoking, alcohol consumption, exercise routine, diet), physical examination (height, weight, body mass index, body temperature, respiration, pulse, blood pressure), and disease characteristics (such as tumor grading and staging).

Among the 21 studies, the most popular statistical method was LR, accounting for 85.7%. As for machine learning, random forests (RF) accounted for 42.9%, followed by support vector machines (38.1%) and XGBoost (33.3%). 90.5% (19/21) of studies showed that the integration models outperformed the individual models. Only 8 studies underwent external validation. The reported metrics’ values of the included studies were shown in Tables S2 to S6. AUROC of integration models surpassed 0.75 and performed better than both statistical methods and machine learning, but there was only a relatively small difference. Two studies [30,36] using multiple integrating strategies found that stacking method appeared slightly superior to weighted averaging (AUROC, 0.820 versus 0.812) and majority voting (AUROC, 0.989 versus 0.930). However, the *P* value of the difference of these methods was not given in the 2 studies. The accuracy and *F*-measure of integration models also surpassed those of statistical methods and machine learning; nevertheless, the differences among most studies were also small (see Tables S3 and S4).

## Discussion

The review found that current research on integrating machine learning into statistical methods for clinical prediction modeling was still relatively limited. Regression models adopted integrating strategies such as simple statistics, weighted statistics, and stacking. For classification models, strategies included majority voting, weighted voting, stacking, and model selection. The integration models could use both high-dimensional or complex variables and traditional clinical features. Despite the limited number of studies, some studies have found that integration models outperformed single models in disease prediction. Stacking method may be suitable in the case of a large number of predictors but required relatively larger amount of training data. Many included studies were lack of complete and clear report of modeling details and result, as well as external validation of models.

Our study found that integration models were primarily applied to the prediction of chronic diseases’ onset and prognosis. This might be related to the global burden and impact of chronic diseases, coupled with the increasing availability of clinical data that offer ample opportunities for prediction. For instance, there were 529 million diabetes patients globally in 2021, and it is projected to reach 1.31 billion by 2050 [40]. Researchers have employed integration models to improve the accuracy of diabetes risk prediction [33]. Additionally, cancer incidence and mortality rates are continuously rising [41]. Integration models could assist medical professionals more accurately assessing cancer patients’ risks and prognoses [18,19]. In the future, integration methods could be considered to expand into other disease fields like neurodegenerative disorders and emerging infectious diseases.

Each integration strategy has its advantages and limitations. The selection of integrating strategies depends on data characteristics, model complexity, application scenarios, and the requirements of prediction goals. For regression models, although the statistical integration is easy to apply and needs less training data, it does not take into account the inherent differences between the different models [31,37]. In contrast, stacking models could better amalgamate the strengths of different models and enhance prediction performance [22,25], and were found suitable when using a large number of predictors. However, the training of each layer requires the use of different data; therefore, stacking models may demand more computational resources and data support than simple statistics [39,42]. Similarly, for classification models, majority voting and weighted voting are straightforward but fail to fully leverage performance differences among different models. Stacking models can learn combination rules from diverse models.

Researchers should be cautious when applying integration methods to different settings. The data in the currently included studies have a low proportion of missing values, which may differ from actual clinical prediction situations. Nevertheless, this study also detailed the corresponding methods of handling missing values, which can be referenced in practical applications. In addition, this study found few instances of overfitting. However, when selecting variables for integration modeling according to own dataset, it is important to be cautious of overfitting.

Results showed that the most common statistical method in integration models was LR, which predicts the target variable by establishing a linear relationship model and provides insights into the weights and influences among variables [43]. Machine learning algorithms such as RF and XGBoost also have widespread application in clinical prediction. RF operates by constructing numerous decision trees during training and is a collective learning approach used for classification, regression, and other tasks. XGBoost predicts in the form of an ensemble of weak prediction models, improving model performance through continuously iterative iterations [44]. However, these 2 methods still retain complexities, operational challenges, and difficulties in interpretation. During the integrating process, applying too many models simultaneously increases the difficulty and computation cost of the implementation. We should select the optimal type and quantity of statistical methods and machine learning for integration according to data characteristics and problem complexity.

At present, integration models have been less applied in clinical practice. Compared to individual methods, integration models may entail greater complexity in operation, higher demands on machinery and clinicians who may lack statistical basis, as well as increased investment. Moreover, the increase in training steps may also raise concerns about data security. Besides, the majority of studies lack complete reporting of modeling methodology and have not undergone external validation, thereby leaving their practical value insufficiently substantiated, which is also the direction of urgent improvement in the future. We also found that in most scenarios, there was a relatively small difference in performance between the integration models and statistical methods or machine learning. Comprehensively considering the application complexity, cost, and benefit, the integration models might not always be the optimal choice. In addition, the common model evaluation metrics currently used, such as AUROC, still lack relevance to clinical practice [45], making it difficult to comprehensively assess the clinical value and cost-effectiveness of different models. Developing and applying comprehensive model evaluation tools and ways is also a direction for further advancement.

## Conclusions

This study comprehensively reviewed the current status of integrating machine learning into traditional statistical methods in clinical prediction. Existing studies indicated the potential of integration modeling in enhancing clinical prediction accuracy and robustness. In practical application, it is necessary to choose appropriate integration strategies based on the specific scenarios and requirements. Future research needs to further address the technical challenges of integration and improve reporting standards, developing more efficient and generalizable integration models tailored to different scenarios, in order to effectively serve clinical decision-making.

## Data Availability

This study is a review, using only data extracted from previous studies.

## Supplementary Materials

Supplementary 1 Search strategy Tables S1 to S6
