# Supplementary material for: Integrating Machine Learning into Statistical Methods in Disease Risk Prediction Modeling: A Systematic Review
Source: Health Data Sci. 2024 Jul 23;4:0165. doi: 10.34133/hds.0165 (PMC11266123; doi:10.34133/hds.0165)
Supplement: Supplementary 1 — Search strategy Tables S1 to S6 [file hds.0165.f1.docx]

**Supplementary Material**

**Search strategy**

**Search strategy for PubMed (3836 items)**

1. (((((((((((((((((((artificial intelligence[Title/Abstract]) OR (artificial intelligence[MeSH Terms])) OR (computational intelligence[Title/Abstract])) OR (computational intelligence[MeSH Terms])) OR (machine intelligence[Title/Abstract])) OR (machine intelligence[MeSH Terms])) OR (machine learning[Title/Abstract])) OR (machine learning[MeSH Terms])) OR (deep learning[Title/Abstract])) OR (deep learning[MeSH Terms])) OR (reinforcement learning[Title/Abstract])) OR (reinforcement learning[MeSH Terms])) OR (neutral network*[Title/Abstract])) OR (neutral network*[MeSH Terms])) OR (ensemble learning[Title/Abstract])) OR (ensemble learning[MeSH Terms])) OR (supervised learning[Title/Abstract])) OR (supervised learning[MeSH Terms])) OR (unsupervised learning[Title/Abstract])) OR (unsupervised learning[MeSH Terms])

2. Search: ((((((disease prediction[Title/Abstract]) OR (risk prediction[Title/Abstract])) OR (prediction model[Title/Abstract])) OR (diagnosis model[Title/Abstract])) OR (prognostic prediction[Title/Abstract])) OR (prognostic model[Title/Abstract]))

3. Search: ((((((((((((((((((((receiver operating characteristic*[Title/Abstract]) OR (ROC curve*[Title/Abstract])) OR (ROC analaysis[Title/Abstract])) OR (ROC analayses[Title/Abstract])) OR (area under curve*[Title/Abstract])) OR (AUC[Title/Abstract])) OR (C-statistic*[Title/Abstract])) OR (specificity[Title/Abstract])) OR (sensitivity[Title/Abstract])) OR (R2[Title/Abstract])) OR (accuracy[Title/Abstract])) OR (AIC[Title/Abstract])) OR (BIC[Title/Abstract])) OR (QAIC[Title/Abstract])) OR (HQ[Title/Abstract])) OR (DIC[Title/Abstract])) OR (Hosmer-Lemeshow test*[Title/Abstract])) OR (calibration curve*[Title/Abstract])) OR (calibration plot*[Title/Abstract])) OR (expected calibration error[Title/Abstract])) OR (ECE[Title/Abstract])

1. #1 AND #2 AND #3

**Search strategy for Web of Science (2401 items)**

1. (((((((((TI=(artificial intelligence)) OR TI=(computational intelligence)) OR TI=(machine intelligence)) OR TI=(machine learning)) OR TI=(deep learning)) OR TI=(reinforcement learning)) OR TI=(neutral network*)) OR TI=(ensemble learning)) OR TI=(supervised learning)) OR TI=(unsupervised learning)

2. (((((TI=(disease prediction)) OR TI=(risk prediction)) OR TI=(prediction model)) OR TI=(diagnosis model)) OR TI=(prognostic model)) OR TI=( prognostic prediction)

3. ((((((((((((((((((((AB=(receiver operating characteristic*)) OR AB=(ROC curve*)) OR AB=(ROC analaysis)) OR AB=(ROC analayses)) OR AB=(area under curve*)) OR AB=(AUC)) OR AB=(C-statistic*)) OR AB=(sensitivity)) OR AB=(specificity)) OR AB=(R2)) OR AB=(Accuracy)) OR AB=(AIC)) OR AB=(BIC)) OR AB=(QAIC)) OR AB=(HQ)) OR AB=(DIC)) OR AB=(Hosmer-Lemeshow test*)) OR AB=(calibration curve*)) OR AB=(calibration plot*)) OR AB=(expected calibration error)) OR AB=(ECE)

1. #1 AND #2 AND #3

**Search strategy for Embase (1396 items)**

1. 'receiver operating characteristic*':ab,ti OR 'roc curve*':ab,ti OR 'roc analaysis':ab,ti OR 'roc analayses':ab,ti OR 'area under curve*':ab,ti OR auc:ab,ti OR 'c statistic*':ab,ti OR sensitivity:ab,ti OR specificity:ab,ti OR r2:ab,ti OR accuracy:ab,ti OR aic:ab,ti OR bic:ab,ti OR qaic:ab,ti OR hq:ab,ti OR dic:ab,ti OR 'hosmer-lemeshow test*':ab,ti OR 'calibration curve*':ab,ti OR 'calibration plot*':ab,ti OR 'expected calibration error':ab,ti OR ece:ab,ti

2. 'disease prediction':ti OR 'risk prediction':ti OR 'prediction model':ti OR 'diagnosis model':ti OR 'prognostic prediction':ti OR 'prognostic model':ti

3. 'artificial intelligence'/exp OR 'computational intelligence'/exp OR 'machine intelligence'/exp OR 'machine learning'/exp OR 'deep learning'/exp OR 'reinforcement learning'/exp OR 'neutral network' OR 'ensemble learning'/exp OR 'supervised learning'/exp OR 'unsupervised learning'/exp OR 'artificial intelligence':ti OR 'computational intelligence':ti OR 'machine intelligence':ti OR 'machine learning':ti OR 'deep learning':ti OR 'reinforcement learning':ti OR 'neutral network*':ti OR 'ensemble learning':ti OR 'supervised learning':ti OR 'unsupervised learning':ti

1. #1 AND #2 AND #3

**Search strategy for CNKI (615 items)**

(TI=人工智能 OR TI=机器学习 OR TI=深度学习 OR TI=监督学习 OR TI=强化学习 OR TI=神经网络 OR TI=集成学习) AND (TI=发病风险 OR TI=风险预测 OR TI=预测模型 OR TI=疾病预后 OR TI=诊断模型) AND (AB=受试者工作特征曲线 OR AB=AUC OR AB=ROC OR AB=C-statistic OR AB=灵敏度 OR AB=敏感度 OR AB=真阳性率 OR AB=特异度 OR AB=真阴性率 OR AB=R2 OR AB=决定系数 OR AB=准确率 OR AB=正确率 OR AB=AIC OR AB=BIC OR AB=QAIC OR AB=HQ OR AB=DIC OR AB=Hosmer-Lemeshow检验 OR AB=校准曲线 OR AB=校正曲线 OR AB=预期校准误差 OR AB=期望校准误差 OR AB=ECE)

**Search strategy for WanFang (386 items)**

（题名:(人工智能) or 题名:(机器学习) or 题名:(深度学习) or 题名:(监督学习) or 题名:(强化学习) or 题名:(集成学习) or 题名:(神经网络)) and (题名:(发病风险) or 题名:(风险预测) or 题名:(预测模型) or 题名:(疾病预后) or 题名:(诊断模型)) and (摘要:(受试者工作特征曲线) or 摘要:(AUC) or 摘要:(ROC) or 摘要:(C-statistic) or 摘要:(灵敏度) or 摘要:(敏感度) or 摘要:(真阳性率) or 摘要:(特异度) or 摘要:(真阴性率) or 摘要:(R2) or 摘要:(决定系数) or 摘要:(准确率) or 摘要:(正确率) or 摘要:(AIC) or 摘要:(BIC) or 摘要:(QAIC) 摘要:(HQ) or 摘要:(DIC) or 摘要:(Hosmer-Lemeshow检验) or 摘要:(校准曲线) or 摘要:(校正曲线) or 摘要:(预期校准误差) or 摘要:(期望校准误差) or 摘要:(ECE))

**Search strategy for VIP (582 items)**

(T=人工智能 OR T=机器学习 OR T=深度学习 OR T=监督学习 OR T=强化学习 OR T=神经网络 OR T=集成学习) AND (T=发病风险 OR T=风险预测 OR T=预测模型 OR T=疾病预后 OR T=诊断模型) AND (R=受试者工作特征曲线 OR R=AUC OR R=ROC OR R=C-statistic OR R=灵敏度 OR R=敏感度 OR R=真阳性率 OR R=特异度 OR R=真阴性率 OR R=R2 OR R=决定系数 OR R=准确率 OR R=正确率 OR R=AIC OR R=BIC OR R=QAIC OR R=HQ OR R=DIC OR R=ECE OR R=Hosmer-Lemeshow检验 OR R=校准曲线 OR R=校正曲线 OR R=预期校准误差 OR R=期望校准误差)

**Search strategy for SinoMed (617 items)**

(("Hosmer-Lemeshow检验"[摘要:智能] OR "校准曲线"[摘要:智能] OR "校正曲线"[摘要:智能] OR "预期校准误差"[摘要:智能] OR "期望校准误差"[摘要:智能] OR "ECE"[摘要:智能]) OR ("R2"[摘要:智能] OR "决定系数"[摘要:智能] OR "准确率"[摘要:智能] OR "正确率"[摘要:智能] OR "AIC"[摘要:智能] OR "BIC"[摘要:智能] OR "QAIC"[摘要:智能] OR "HQ"[摘要:智能] OR "DIC"[摘要:智能]) OR ("受试者工作特征曲线"[摘要:智能] OR "C-statistic"[摘要:智能] OR "ROC"[摘要:智能] OR "AUC"[摘要:智能] OR "灵敏度"[摘要:智能] OR "敏感度"[摘要:智能] OR "真阳性率"[摘要:智能] OR "特异度"[摘要:智能] OR "真阴性率"[摘要:智能])) AND ("发病风险"[摘要:智能] OR "预测模型"[摘要:智能] OR "疾病预后"[摘要:智能] OR "诊断模型"[摘要:智能] OR "风险预测"[摘要:智能]) AND ("人工智能"[摘要:智能] OR "机器学习"[摘要:智能] OR "深度学习"[摘要:智能] OR "监督学习"[摘要:智能] OR "强化学习"[摘要:智能] OR "无监督学习"[摘要:智能] OR "神经网络"[摘要:智能] OR "集成学习"[摘要:智能])

**Table S1: Basic information of the included studies.**

| **First author, Publication Year** | **Data type** | **Data source** | **Target population** | **Outcome** | **Prediction interval or follow-up time** | **Types of integrating** |
| --- | --- | --- | --- | --- | --- | --- |
| **Regression model** |  |  |  |  |  |  |
| Lippmann 1997 [18]^a^ | Multicenter | National registration database | Patients who underwent coronary artery bypass grafting | Death | NA | Simple averaging |
| Lv 2011 [19]^a^ | Single center | Data from hospitals | Patients with colorectal cancer | Liver metastasis | NA | Simple averaging |
| Dessie 2022 [20]^a^ | NA | TCGA dataset | Patients with lung adenocarcinoma | Death | >30 d | Simple averaging |
| Pai 2022 [21]^b^ | Single center | Data from hospitals | Patients requiring mechanical ventilation | Acute respiratory distress syndrome | 48 h | Simple averaging or maximum |
| Basu 2019 [22]^a^ | Multicenter | Administrative claims data; US Census, US CDC, and related federal data | Patients with T2DM | Uncontrolled diabetes | NA | Weighted combination of all the models |
| Gao 2020 [23]^a^ | Single center | Data from hospitals | COVID-19 patients | Death | 23 d | Weighted combination of all the models |
| Torquati 2022 [24]^a^ | Multicenter | National registration database | Patients who underwent elective laparoscopic gastric bypass or laparoscopic sleeve gastrectomy | Readmission | 30 d | Weighted combination of all the models |
| Warden 2021 [25]^b^ | Multicenter | Medicare administrative claims data | General population | Parkinson disease | NA | Stacking (LR as the meta-classifier) |
| Zeng 2021 [26]^a^ | Multicenter | Data from hospitals | ICU patients with sepsis | Death | 70 h | Stacking (LR as the meta-classifier) |
| Khera 2021 [27]^a^ | Multicenter | National registration database | AMI hospitalization | Death from any cause | During hospitalization | Stacking (XGBoost as the meta-classifier) |
| Liu 2021a [28]^a^ | Single center | Data from hospitals | Breast cancer patients | Disease progress after initial diagnosis | 5 y; 10 y | Stacking (XGBoost as the meta-classifier) |
| Liu 2021b [29]^b^ | Single center | Data from hospitals | Patients with pulmonary nodules | Lung cancer | NA | Stacking (LR as the meta-classifier) |
| Lee 2022 [30]^a^ | Multicenter | Health insurance data | General population | Pancreatic cancer | NA | Stacking (LR as the meta-classifier); Averaging |
| Fan 2021 [31]^a^ | Single center | Data from hospitals | Patients with diffuse large B-cell lymphoma | Death | 2 y | Stacking (LR as the meta-classifier); Simple averaging, Weighted averaging |
| **Classification model** |  |  |  |  |  |  |
| Rustam 2022 [32]^b^ | Multicenter | Public data | General population | Cardiovascular disease | NA | Majority voting |
| Bashir 2015 [33]^b^ | Multicenter | Public data | General population | Heart disease | NA | Weighted voting (F-measure as the weight) |
| Wu 2018 [34]^a^ | NA | Public data | General population | Diabetes | NA | Stacking (LR was used in the second level) |
| Kong 2021 [35]^a^ | Multicenter | Data from hospitals | Patients treated with peritoneal dialysis | Prolonged length of stay | NA | Stacking (LR as the meta-classifier) |
| Zhang 2021 [36]^a^ | Single center | Data from hospitals | Patients diagnosed with histology-positive Cushing’s disease | Immediate remission after transsphenoidal surgery | Predict the postoperative situation before operation | Stacking (LR as the meta-classifier) |
| Dritsas 2022 [37]^a^ | NA | Public data | Adults | Stroke | NA | Stacking (LR as the meta-classifier); Majority voting |
| Chun 2021 [38]^a^ | Multicenter | China Kadoorie Biobank | Adults without prior history of stroke | Stroke | <9y; 0～3y; 3～6y; 6～9y | New method was used to select the final model when two models disagreed. |

^a^ Prognostic model. ^b^ Diagnostic model. TCGA, The Cancer Genome Atlas Lung Adenocarcinoma; T2DM, Type 2 diabetes mellitus; COVID-19, Corona Virus Disease 2019; LR, Logistic Regression; ICU, Intensive Care Unit; AMI, Acute Myocardial Infarction; XGBoost, eXtreme Gradient Boosting.

**Table S2: AUROC of models among the included studies.**

| **First Author, Publication Year** | **Statistical methods** | |  | **Machine learning** | |  | **Integration model** | |
| --- | --- | --- | --- | --- | --- | --- | --- | --- |
|  | **Method** | **AUROC** |  | **Method** | **AUROC** |  | **Method** | **AUROC** |
| **Regression** |  |  |  |  |  |  |  |  |
| Lippmann 1997 [18] | LR | 0.762 |  | MLP | 0.761 |  | Simple averaging | **0.764** |
| Lv 2011 [19] | LR | 0.870 |  | AdaBoost | 0.878 |  | Simple averaging | **0.921** |
| Dessie 2022 [20] | LR | 0.739 |  | NN | **0.842** |  | Simple averaging | 0.812 |
| Pai 2022 [21] | LR | 0.902 |  | XGBoost; RF | 0.910 |  | Average | **0.912** |
|  |  |  |  |  |  |  | Maximum | **0.912** |
| Basu 2019 [22] | LR with elastic net regularization | 0.685 |  | RF | **0.928** |  | Weighted combination | 0.924 |
| Gao 2020 [23] | LR | 0.961 |  | NN | **0.962** |  | Weighted combination | **0.962** |
| Torquati 2022 [24] | LR | 0.650 |  | GAM; XGBoost | NA |  | Weighted combination | **0.674** |
| Warden 2021 [25] | Penalized LR | 0.827 |  | RF | 0.826 |  | Stacking | **0.835** |
| Zeng 2021 [26] | LR | 0.789 |  | XGBoost | 0.814 |  | Stacking | **0.815** |
| Khera 2021 [27] | LR | 0.888 |  | XGBoost | 0.898 |  | Stacking | **0.899** |
| Liu 2021 [28] | Cox regression | 0.715 |  | XGBoost | 0.777 |  | Stacking | **0.782** |
| Liu 2021 [29] | LR | NA |  | CNN | 0.907 |  | Stacking | **0.916** |
| Lee 2022 [30] | LR | **0.770** |  | MLP; RF; SVM; KNN; XGBoost; CART; Bayes | NA |  | Averaging | 0.760 |
|  |  |  |  |  |  |  | Stacking | 0.755 |
| Fan 2021 [31] | LR | 0.803 |  | FNN | 0.813 |  | Simple averaging | 0.811 |
|  |  |  |  |  |  |  | Weighted averaging | 0.812 |
|  |  |  |  |  |  |  | Stacking | **0.820** |
| **Classification** |  |  |  |  |  |  |  |  |
| Kong 2021 [35] | LR | 0.742 |  | RF | 0.756 |  | Stacking | **0.757** |
| Zhang 2021 [36] | LR | 0.701 |  | GBDT | 0.734 |  | Stacking | **0.743** |
| Dritsas 2022 [37] | LR | 0.877 |  | RF | 0.986 |  | Majority voting | 0.930 |
|  |  |  |  |  |  |  | Stacking | **0.989** |

Bold indicates the highest performance value within the study. LR, Logistic Regression; MLP, Multi-Layer Perceptron; AdaBoost, Adaptive Boosting; NN, Neural Network; XGBoost, eXtreme Gradient Boosting; RF, Random Forest; GAM, generalized additive models; SVM, Support Vector Machine; KNN, K-Nearest Neighbor; CART, Classification and Regression Trees; FNN, Feedforward Neural Network; GBDT, Gradient Boosting Decision Tree.

**Table S3: Accuracy of models among the included studies.**

| **First Author, Publication Year** | **Statistical methods** | |  | **Machine learning** | |  | **Integration model** | |
| --- | --- | --- | --- | --- | --- | --- | --- | --- |
|  | **Method** | **Accuracy** |  | **Method** | **Accuracy** |  | **Method** | **Accuracy** |
| **Regression** |  |  |  |  |  |  |  |  |
| Pai 2022 [21] | LR | 83.2% |  | XGBoost | 84.8% |  | Simple averaging | **87.1%** |
|  |  |  |  |  |  |  | Maximum | 82.1% |
| Basu 2019 [22] | LR with elastic net regularization | 68.4% |  | RF | **90.6%** |  | Weighted combination | 89.8% |
| Gao 2020 [23] | LR | 92.1% |  | SVM | **92.4%** |  | Weighted combination | **92.4%** |
| Lee 2022 [30] | LR | 70.0% |  | MLP; RF; SVM; KNN; XGBoost; CART; Bayes | NA |  | Averaging | **70.5%** |
|  |  |  |  |  |  |  | Stacking | 70.0% |
| **Classification** |  |  |  |  |  |  |  |  |
| Rustam 2022 [32] | LR | 90.0% |  | SVM; SGDC | 88.0% |  | Majority voting | **92.0%** |
| Bashir 2015 [33] | Linear regression | 82.9% |  | SVM | 79.7% |  | Weighted voting | **83.0%** |
| Wu 2018 [33] | LR | 72.0% |  | K-means | NA |  | Stacking | **90.7%** |
| Kong 2021 [35] | LR | 67.5% |  | RF | 69.1% |  | Stacking | **69.5%** |
| Zhang 2021 [36] | LR | 73.2% |  | RF | 74.6% |  | Stacking | 74.6% |
| Dritsas 2022 [37] | LR | 79.0% |  | RF | **97.0%** |  | Majority voting | 93.0% |
| Chun 2021 (Men) [38] | Cox regression | 75.0% |  | GBT | 75.0% |  | Model-selection | 76.0% |
| Chun 2021 (Women) [38] | Cox regression | 79.0% |  | GBT | 77.0% |  | Model-selection | **80.0%** |

Bold indicates the highest performance value within the study. LR, Logistic Regression; XGBoost, eXtreme Gradient Boosting; RF, Random Forest; SVM, Support Vector Machine; MLP, Multi-Layer Perceptron; KNN, K-Nearest Neighbor; CART, Classification and Regression Trees; SGDC, Stochastic Gradient Descent Classifier; GBT, gradient boosted trees.

**Table S4: F-measure of models among the included studies.**

| **First Author, Publication Year** | **Statistical methods** | |  | **Machine learning** | |  | **Integration model** | |
| --- | --- | --- | --- | --- | --- | --- | --- | --- |
|  | **Method** | ***F*-measure** |  | **Method** | ***F*-measure** |  | **Method** | ***F*-measure** |
| **Regression** |  |  |  |  |  |  |  |  |
| Gao 2020 [23] | LR | 0.593 |  | SVM | **0.638** |  | Weighted combination | 0.600 |
| **Classification** |  |  |  |  |  |  |  |  |
| Rustam 2022 [32] | LR | 0.900 |  | SVM; SGDC | 0.870 |  | Majority voting | **0.920** |
| Bashir 2015 [33] | Linear regression | 0.781 |  | NB | 0.772 |  | Weighted voting | **0.809** |
| Dritsas 2022 [37] | LR | 0.791 |  | RF | 0.966 |  | Majority voting | 0.930 |
|  |  |  |  |  |  |  | Stacking | **0.974** |

Bold indicates the highest performance value within the study. LR, Logistic Regression; SVM, Support Vector Machine; SGDC, Stochastic Gradient Descent Classifier; NB, Naïve Bayes; RF, Random Forest.

**Table S5: Sensitivity of models among the included studies.**

| **First Author, Publication Year** | **Statistical methods** | |  | **Machine learning** | |  | **Integration model** | |
| --- | --- | --- | --- | --- | --- | --- | --- | --- |
|  | **Method** | **Sensitivity** |  | **Method** | **Sensitivity** |  | **Method** | **Sensitivity** |
| **Regression** |  |  |  |  |  |  |  |  |
| Pai 2022 [21] | LR | 79.1% |  | XGBoost | 80.9% |  | Average | 67.6% |
|  |  |  |  |  |  |  | Maximum | **84.9%** |
| Basu 2019 [22] | LR with elastic net regularization | 25.6% |  | RF | **68.5%** |  | Weighted combination | 65.6% |
| Gao 2020 [23] | LR | 45.0% |  | SVM | **57.9%** |  | Weighted combination | 45.0% |
| Warden 2021 [25] | Penalized LR | 67.2% |  | RF | 66.3% |  | Stacking | **72.9%** |
| Khera 2021 [27] | LR | 47.0% |  | XGBoost | 45.0% |  | Stacking | 43.0% |
| Lee 2022 [30] | LR | 70.0% |  | MLP; RF; SVM; KNN; XGBoost; CART; Bayes | NA |  | Averaging | **70.5%** |
|  |  |  |  |  |  |  | Stacking | **70.5%** |
| **Classification** |  |  |  |  |  |  |  |  |
| Rustam 2022 [32] | LR | 90.0% |  | SVM | 88.0% |  | Majority voting | **92.0%** |
| Bashir 2015[33] | Linear regression | 87.9% |  | SVM | **91.8%** |  | Weighted voting | 89.2% |
| Kong 2021 [35] | LR | 68.3% |  | RF | 68.6% |  | Stacking | 68.0% |
| Dritsas 2022 [37] | LR | 79.1% |  | RF | 96.6% |  | Majority voting | 93.0% |
|  |  |  |  |  |  |  | Stacking | **97.4%** |
| Chun 2021 (Men) [38] | Cox regression | 76.0% |  | GBT | **80.0%** |  | Model-selection | 76.0% |
| Chun 2021 (Women) [38] | Cox regression | 68.0% |  | GBT | **74.0%** |  | Model-selection | 67.0% |

Bold indicates the highest performance value within the study. LR, Logistic Regression; XGBoost, eXtreme Gradient Boosting; RF, Random Forest; SVM, Support Vector Machine; MLP, Multi-Layer Perceptron; KNN, K-Nearest Neighbor; CART, Classification and Regression Trees; GBT, gradient boosted trees.

**Table S6: Specificity of models among the included studies.**

| **First Author, Publication Year** | **Statistical methods** | |  | **Machine learning** | |  | **Integration model** | |
| --- | --- | --- | --- | --- | --- | --- | --- | --- |
|  | **Method** | **Specificity** |  | **Method** | **Specificity** |  | **Method** | **Specificity** |
| **Regression** |  |  |  |  |  |  |  |  |
| Pai 2022 [21] | LR | 84.5% |  | XGBoost | 86.1% |  | Average | **93.4%** |
|  |  |  |  |  |  |  | Maximum | 81.2% |
| Basu 2019 [22] | LR with elastic net regularization | 90.1% |  | RF | **94.6%** |  | Weighted combination | 94.2% |
| Gao 2020 [23] | LR | 98.1% |  | NN | **98.9%** |  | Weighted combination | 98.3% |
| Warden 2021 [25] | Penalized LR | **92.6%** |  | RF | 82.8% |  | Stacking | 79.6% |
| Khera 2021 [27] | LR | 97.0% |  | XGBoost | **98.0%** |  | Stacking | **98.0%** |
| Lee 2022 [30] | LR | 70.0% |  | MLP; RF; SVM; KNN; XGBoost; CART; Bayes | NA |  | Averaging | **71.0%** |
|  |  |  |  |  |  |  | Stacking | **71.0%** |
| **Classification** |  |  |  |  |  |  |  |  |
| Bashir 2015 [33] | Linear regression | 77.2% |  | QDA | **82.9%** |  | Weighted voting | 74.0% |
| Kong 2021 [35] | LR | 67.1% |  | SVM | 69.0% |  | Stacking | **70.1%** |
| Chun 2021 (Men) [38] | Cox regression | 75.0% |  | GBT | 74.0% |  | Model-selection | **76.0%** |
| Chun 2021 (Women) [38] | Cox regression | 80.0% |  | GBT | 78.0% |  | Model-selection | **81.0%** |

Bold indicates the highest performance value within the study. LR, Logistic Regression; XGBoost, eXtreme Gradient Boosting; RF, Random Forest; NN, Neural Network; MLP, Multi-Layer Perceptron; SVM, Support Vector Machine; KNN, K-Nearest Neighbor; CART, Classification and Regression Trees; QDA, Quadratic discriminant analysis; GBT, gradient boosted trees.
